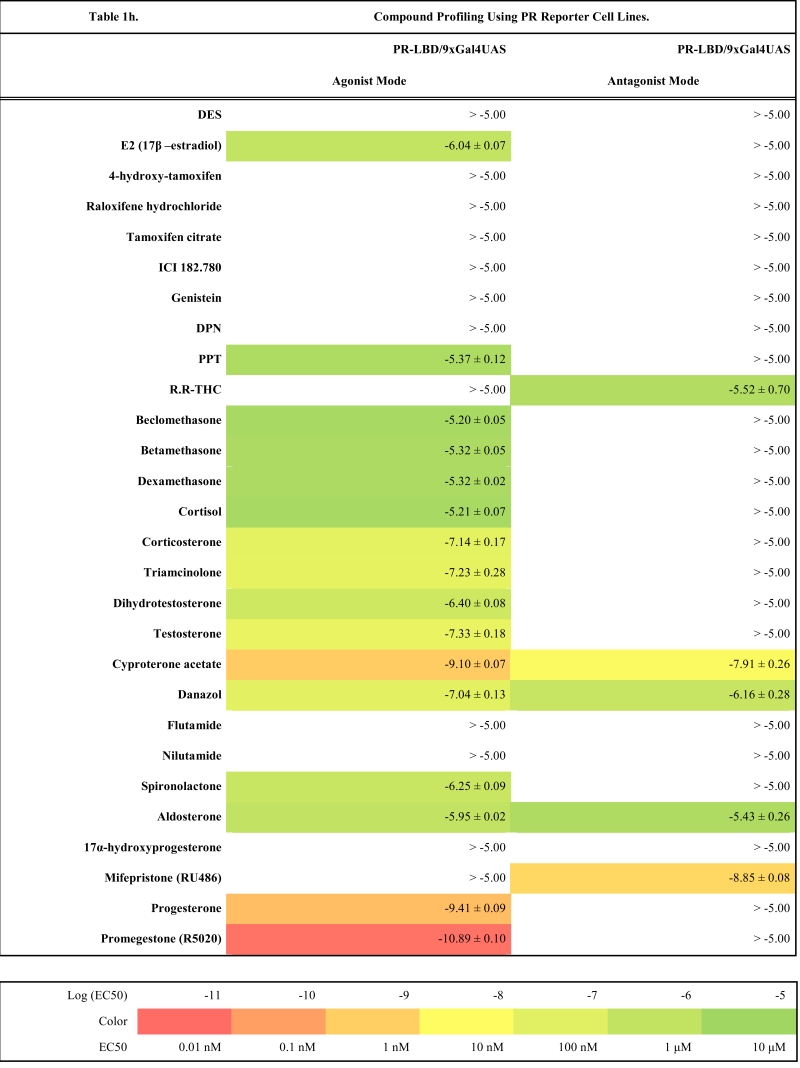# Two Panels of Steroid Receptor Luciferase Reporter Cell Lines for Compound Profiling

**DOI:** 10.2174/138620711795222446

**Published:** 2011-05

**Authors:** David Sedlák, Aileen Paguio, Petr Bartůněk

**Affiliations:** 1 Center for Chemical Genetics & CZ-OPENSCREEN, Institute of Molecular Genetics, v.v.i., Academy of Sciences of the Czech Republic, Vídeňská 1083, 142 20 Prague, Czech Republic; 2 Promega Corporation, 2800 Woods Hollow Road Madison, WI 53711 USA

**Keywords:** U2OS, cell-based luciferase reporter assay, ligand binding domain, Gal4, nuclear hormone receptor, steroid receptor, HTS, drug discovery.

## Abstract

Steroid hormone receptors represent a major target in drug discovery. As ligand inducible transcription factors, their activity can be modulated by small lipophilic molecules. Here we describe two panels of potent and selective luciferase reporter cell lines based on cells with low endogenous steroid receptor activity (U2OS). The panels contain reporter cell lines for estrogen receptors α and β, androgen, glucocorticoid, mineralocorticoid, and progesterone receptors. In the first panel, the activation of either synthetic, steroid response elements containing promoter or viral promoter is mediated by full-length steroid receptors. The second panel is based on the expression of the chimeric receptor, which was created by the replacement of the N-terminal part of the molecule by Gal4 DBD and that binds to multiple UAS sites in the reporter promoter. Both panels were extensively characterized by profiling 28 ligands in dose response manner in agonist and antagonist mode. We have analyzed and compared the responses to tested ligands from both panels and concluded that in general both systems generated similar qualitative response in terms of potency, efficacy, partial agonism/antagonism, mixed agonistic/antagonistic profiles and the rank of potencies was well conserved between both panels. However, we have also identified some artifacts introduced by the Gal4/LBD reporter assays in contrast to their full-length receptor reporter counterparts. Keeping in mind the advantages and drawbacks of each reporter format, these cell lines represent powerful and selective tools for profiling large compound libraries (HTS) and for detailed study of mechanisms by which compounds exert their biological effects.

## INTRODUCTION

Steroid hormone receptors are ligand-inducible transcription factors belonging to nuclear hormone receptor superfamilly and include two receptors for estrogens (ERα and ERβ), androgen receptor (AR), glucocorticoid receptor (GR), mineralocorticoid receptor (MR) and progesterone receptor (PR) [1]. They control essential physiological, developmental, reproductive and metabolic processes. In addition to their indisputable numerous roles in the physiology of healthy organisms, a growing body of work indicates they also represent an important pharmaceutical target in number of diseases including cancer. For instance, ERα and PR are important targets in the breast cancer [2, 3], while AR is the major therapeutic target in prostate tumors [4]. Ligands antagonizing natural hormone ligands for these receptors with reduced side effects are intensively sought as potential drugs for cancer treatment. On the other hand anti-inflammatory properties of GR agonists make this group of ligands indispensable in combating inflammation associated with many diseases [5]. Finally, emerging evidence for the tumor suppressive functions of ERβ in many cancers like breast [6], colon [7], ovarian [8] and prostate [9] cancer make the ERβ selective agonists promising drugs for the future treatment of these tumors.

Steroid hormone receptor activity can be regulated in many different ways including posttranslational modifications resulting from cell signaling activity, for example phosphorylation of target proteins. Expression of different set of coactivator or corepressor molecules and the activity of other transcription factors can be responsible for different transcriptional regulation by steroid receptors in different cellular contexts [10]. However, small lipophilic molecules acting as ligands for these receptors are the main and the most powerful means to modulate steroid receptor activity. In the process of drug and biologically active compound discovery, different approaches are commonly used. These include variety of biochemical assays where the main criterion for identification of active compounds is the binding affinity of the compound to the receptor. Although this approach generates unambiguous data, it provides only very limited information about qualitative changes that the compound induces in the receptor conformation and therefore is difficult to assess the effect of the compound on the transcription of target genes by the receptor. Cell-based reporter assays overcome this limitation and provide more complex information about the compound-receptor interaction and enrich it by reflecting other dimensions of steroid receptor activity such as promoter-receptor interactions, cofactor-receptor recruitment and finally crosstalk with other cellular pathways. Cell-based reporter assays provide rich and complex information that makes them one of the most relevant and important assays for compound profiling and drug discovery.

Several luciferase reporter systems have been developed recently using different cell lines [11-14]. Here we report on a development of two panels of U2OS-based luciferase steroid receptor reporter cell lines using two different reporter formats. In the first format the activity of full-length exogenous steroid receptor is monitored by reporter vector containing synthetic promoter with multimerized responsive elements or viral LTR promoter upstream of the *luc2* gene. The second format relies on the chimeric steroid receptor where the N-terminal part of the receptor containing AF-1 and DNA binding domain (DBD) was replaced by the DBD from the yeast transcription factor Gal4. This construct was cotransfected to the cells together with reporter vector containing 9 copies of Gal4 Upstream Activator Sequences (UAS) coupled to minimal promoter upstream of the *luc2P *gene coding for the luciferase with PEST destabilizing sequence (pGL4.35 [*luc2P*/9XGAL4UAS/Hygro]). This system was described recently, and its utility was illustrated by preparation of ERα and GR reporter assays in HEK293 cells [13]. In this work we extended this system to all members of the steroid receptor family and built a panel of selective reporter cell-lines in U2OS cells. Both of these formats use the latest generation of pGL4 reporter vector backbone [15].

## MATERIALS AND METHODS

### Compounds and Reagents

Diethylstilbestrol (DES), 17β-estradiol (E2), 4-hydroxy-tamoxifen (4-OHT), raloxifene hydrochloride, tamoxifen citrate, ICI 182.780, genistein, (R,R)-cis-diethyltetrahydro-2,8-chrysenediol (R,R-THC), beclomethasone, betamethasone, dexamethasone, cortisol, triamcinolone, 5α-androstan-17β-ol-3-one (DHT, dihydrotestosterone), testosterone, cyproterone acetate, danazol, flutamide, nilutamide, spironolactone, aldosterone, corticosterone, 17α-hydroxyprogesterone, mifepristone (RU486), progesterone were purchased from Sigma-Aldrich (St. Louis, MO, USA); DPN and propyl pyrazole triol (PPT) were from Tocris Bioscience (Bristol, UK), promegestone (R5020) from PerkinElmer (Waltham, MA); Phenol red-free Dulbecco´s Modified Eagle Medium (DMEM) and Fetal Bovine Serum (FBS) were from Invitrogen (Carlsbad, CA, USA); Hyclone Charcoal/Dextran Treated Fetal Bovine Serum (C/D FBS) from Thermo Fisher Scientific Inc. (Waltham, MA, USA). One-Glo Luciferase Assay System was obtained from Promega Corporation (Madison, WI, USA).

### Vectors

#### Expression vectors encoding human full-length steroid receptors:

pcDNA3-hERα: coding sequence for human ERα was excised from the pSG5-HE0 [16] with *EcoRI* and cloned into pcDNA3 expression vector (Invitrogen, Carlsbad, CA, USA) behind the Cytomegalovirus (CMV) promoter. pcDNA3-hERβ: coding sequence for human ERβ was RT-PCR amplified from human hemato-poietic progenitor cells (CFU-C) total RNA with following primers: 5´ CCGCATTTTAGAGAAGGCAAGGCCGG 3´ and 5´ACTGGAGTTCACG CTTCAGCCTGTGACCTC 3´. Amplified DNA was then inserted into the pcDNA3 expression vector. pcDNA3-hGR and pcDNA3-hAR: DNA encoding human GR and AR was ordered from Openbiosystems (Huntsville, AL, USA) (clone ID: 4810424 and 40146997). pcDNA3-hGR was created by transferring the DNA portion encoding GR to BamHI and XhoI sites in the pcDNA3 vector. DNA coding for AR was excised from the parental vector, blunt-ended and inserted into EcoRV site in the pcDNA3 vector. Final constructs were verified by restriction digests and by sequencing. pcDNA3-hMR was described previously [17] and was provided as a gift by Marie-Edith Rafestin-Oblin (INSERM, France).

#### Expression vectors encoding chimeric receptors consisting of Gal4-DBD and of the ligand binding domain (LBD) of the human steroid receptor:

 Creation of pBIND-ERα^G420C^ and pBIND-GR in pFN26A (BIND) vector was described earlier [13].

For pBIND-ERα^wt^, a DNA sequence of ERα-LBD (amino acids 303-595) based on GenBank NM_000125 was synthesized by DNA 2.0 (Menlo Park, CA), and cloned into pFN26A (BIND) *hRluc*-neo (Promega) using *SgfI* and *PmeI* so that the *SgfI* site yields an in-frame protein fusion with GAL4-DBD.

ERβ-LBD was cloned by PCR amplification of the region comprising ERβ-LBD and a piece of the hinge region using previously cloned full-length ERβ as template and following primers: 5´AACAGCGATCGCCCAGGCCTGCC GACTTCGGAAG 3´ and 5´ AGAA GTTTAAACCTGAGA CTGTGGGTTCTGGGAGCC 3´. Similarly AR-LBD was cloned using previously cloned full-length AR as template, and the following primers were used for the amplification of the LBD region: 5´ AACAGCGATCGCCGCCCGGAAGC TGAA GAAACTTGG 3´ and 5´ AGAAGTTTAAACCT GGGTGTGGAAATAGATGGGCTTG 3´. MR-LBD was cloned by RT-PCR from total RNA isolated from HEK293 cells using following primer combination: 5´ AACAGCGA TCGCCCCCTCGGTCAACACAGCACTGG 3´ and 5´ AGAAGTTTAAACCTTCCGGTGGAAGTAGAGCGGC 3´. Plasmid encoding human PR was purchased from Openbiosystems (clone ID: 100016179), and PR-LBD region was PCR amplified using the following primer combination: 5´ AACAGCGATCGCCGAAAGCCAAGCC CTAAGC CAGAG 3´ and 5´ AGAAGTTTAAACCTTTTT ATGAAAGAGAAGGGGTTTCACC 3´. PCR products with steroid receptor LBDs were digested by *SgfI/PmeI* and inserted into *SgfI/PmeI* sites in the pFN26A (BIND) vector. Final constructs were verified by restriction digests and sequencing.

#### Reporter vectors:

pGL4.26-3xERE [*luc2*/Hygro] was created by inserting an oligonucleotide comprising three copies of estrogen responsive elements (ERE) CCAGGTC ACAGTGACCTGAGT in the pGL4.26 [*luc2*/minP/Hygro] reporter vector (Promega, Madison, WI, USA). In this synthetic estrogen responsive promoter the centers of the perfect palindromic inverted repeats are separated by 21 bp from each other. The 3xERE containing oligonucleotide was inserted in the *XhoI/BglII* restriction sites of pGL4.26 [*luc2*/minP/Hygro] reporter vector. pGL4.26-3xGRE [*luc2*/Hygro] was constructed in a similar way by insertion of an oligonucleotide containing three copies of glucocorti-coid responsive elements (GRE) GGTACATTTTGTT CTAGCCAG in the *XhoI/BglII* restriction sites of pGL4.26 [*luc2*/minP/Hygro] reporter vector.

pGL4.35 [luc2P/9XGAL4UAS/Hygro] and pGL4.36 [luc2P/MMTV/Hygro] reporter vectors were described previously [13].

Final constructs were verified by restriction digests and sequencing.

### Generation of Stable Reporter Cell-Lines

The production of stable reporter cell lines for both full-length steroid hormone receptors and chimeric steroid LBD receptors in U2OS cells was done in two steps. In the first step, U2OS cells were transfected with reporter vector alone (pGL4.26-3xERE [*luc2*/Hygro], pGL4.26-3xGRE [*luc2*/ Hygro], pGL4.36 [*luc2P*/MMTV/Hygro] or pGL4.35 [*luc2P*/ 9XGAL4UAS/Hygro]) using PEI 25kDa (PolySciences, Inc., Warrington, PA, USA) transfection. Two days after transfection, selection of stable transfectants was initiated by adding Hygromycin B (Invitrogen, Carlsbad, CA, USA) to the culture medium at final concentration of 100 μg/ml. Cells were grown in the Hygromycin B-containing medium for three weeks, and after that time cells were frozen and were used later for transfection with expression vectors for steroid receptors.

#### Panel of U2OS Steroid receptor-LBD/9xGal4UAS luciferase reporter cell-lines:

U2OS cell line stably transfected with pGL4.35 [*luc2P*/9XGAL4UAS/Hygro] was transfected with expression vectors for chimeric steroid receptors: pBIND-ERα^wt^, pBIND-ERα^G420C^, pBIND-ERβ, pBIND-AR, pBIND-GR, pBIND-MR and pBIND-PR to give rise to the following reporter cell-lines: U2OS-ERα^wt^-LBD/9xGal4UAS, U2OS-ERα^G420C^-LBD/9xGal4UAS, U2OS- ERβ-LBD/9xGal4UAS, U2OS-AR-LBD/9xGal4UAS, U2OS- GR-LBD/9xGal4UAS, U2OS-MR-LBD/9xGal4UAS and U2OS-PR-LBD/9xGal4UAS, respectively. In order to create U2OS-AR-LBD/9xGal4UAS cell line, we cotransfected pBIND-AR vector together with pcDNA3-hAR vector in the 10:1 ratio of the DNA amount in the transfection mixture. Two days after transfection, selection of stable transfectants was initiated by adding G418 and Hygromycin B (Invitrogen, Carlsbad, CA, USA) to the culture medium at final concentration of 250 μg/ml and 100 μg/ml respectively. Cells were propagated in the medium containing antibiotics for at least two months before being frozen and used for compound profiling.

#### Panel of U2OS full-length steroid receptor luciferase reporter cell-lines:

U2OS cell line stably transfected with pGL4.26-3xERE [*luc2*/Hygro] was transfected with expression vectors encoding full-length steroid receptors: pcDNA3-hERα and pcDNA3-ERβ, creating U2OS-ERα/3xERE and U2OS-hERβ/3xERE cell lines, respectively. U2OS cell line stably transfected with pGL4.26-3xGRE [*luc2*/Hygro] was transfected with expression vectors encoding full-length steroid receptors: pcDNA3-hAR and pcDNA3-hGR, creating U2OS-AR/3xGRE and U2OS-GR/3xGRE cell lines, respectively. U2OS cell line stably transfected with pGL4.36 [*luc2P*/MMTV/Hygro] was transfected with expression vectors encoding full-length steroid receptors: pcDNA3-hAR, pcDNA3-hGR and pcDNA3-hMR to generate U2OS-AR/MMTV, U2OS-GR/MMTV, U2OS-MR/MMTV reporter cell lines, respectively. Two days after transfection, selection of stable transfectants was initiated by adding G418 and Hygromycin B (Invitrogen, Carlsbad, CA, USA) to the culture medium at final concentration of 250 μg/ml and 100 μg/ml respectively. Cells were propagated in the antibiotics containing medium for at least two months before being frozen in liquid nitrogen and profiled with different compounds.

### Luciferase Reporter Assay

The luciferase assay was performed in the high throughput screening (HTS) 384-well format using HTS instruments: U2OS stable reporter cell lines were propagated in a monolayer in phenol red-free DMEM supplemented with 10% fetal bovine serum, 4 mM glutamine (Invitrogen, Carlsbad, CA, USA) and penicillin/streptomycin and incubated in a 5% CO_2_ humidified atmosphere at 37°C. Forty-eight hours preceding experiments, growth medium was changed for phenol red-free DMEM supplemented with 4% C/D FBS and 4 mM glutamine (starvation medium). After that time, cells were trypsinized, counted and seeded at a density of 10^4^ cells/well in white opaque cell culture 384-well plates (Corning Inc., NY, USA). Compounds to be tested were serially diluted in DMSO and stored in the polypropylene 384-well plates (Corning Inc., NY, USA). The concentration of serially diluted compounds was in the range of 10 mM to 0.1 nM and each compound was tested at least in 12 different concentrations. They were transferred to cells by JANUS Automated Workstation (PerkinElmer, Inc.) equipped with Pin tool (V&P Scientific, Inc., San Diego, CA, USA). During the Pin tool transfer of compounds from the concentrated DMSO stocks to the culture medium, compounds were 1000x diluted. After incubation for 20 hours, the luciferase activity was determined using One-Glo Luciferase Assay System (Promega, Madison, WI, USA) according to the manufacturer´s protocol. In the antagonist mode, cells were incubated for 30 min with specific agonist at specified concentration prior to Pin tool compound transfer. Luminescence was recorded on EnVision plate reader (PerkinElmer, Inc.) using 1s integration of luminescent signal. The experiment was repeated at least three times. Data were analyzed by GraphPad Prism 5.0 statistical software and LogEC_50_/LogIC_50_ values were generated by fitting data from the luciferase reporter assay by nonlinear regression function (dose response, variable slope). Values are reported as mean ± standard error of the mean (SEM).

## RESULTS

### Creation of U2OS Reporter Cell Lines

To build the full-length steroid receptor reporter system we created two reporter vectors carrying synthetic promoters with three copies of either estrogen responsive element (ERE) or glucocorticoid responsive element (GRE) coupled to a minimal promoter upstream of the coding sequence for the luciferase. ERE and GRE are constituted by the perfect palindromic repeats with a three bp spacing of variable bases. The centers of palindromes are separated by 21 bp from each other, and the distance of the third repeat from the minimal promoter is 50 bp. ERα and ERβ bind to EREs in the promoter of the reporter vector pGL4.26-3xERE [*luc2*/Hygro], while pGL4.26-3xGRE [*luc2*/Hygro] can be used to monitor activity of the remaining steroid receptors: GR, MR, AR and PR. Although all of these receptors can activate the luciferase gene in the presence of the agonist by binding to GRE elements in the reporter vector, the response was not equally efficient in all of these cases. For instance, MR showed only weak transactivation on the 3xGRE-containing synthetic promoter. To overcome these limitations, we combined these receptors with pGL4.36 [*luc2P*/MMTV/Hygro] reporter vector, which contains the enhancer region from the mouse mammary tumor virus long terminal repeat (MMTV LTR) promoter. This region was originally isolated as progesterone and glucocorticoid responsive enhancer [18], but it is also highly inducible by AR and MR. The use of this reporter vector resulted in generally higher dynamic range and higher robustness of the reporter assay. However, the complexity of the viral promoter and presence of responsive elements for other cellular transcription factor add a higher probability of cross-talk with different signaling pathways.

**Fig. (1):**
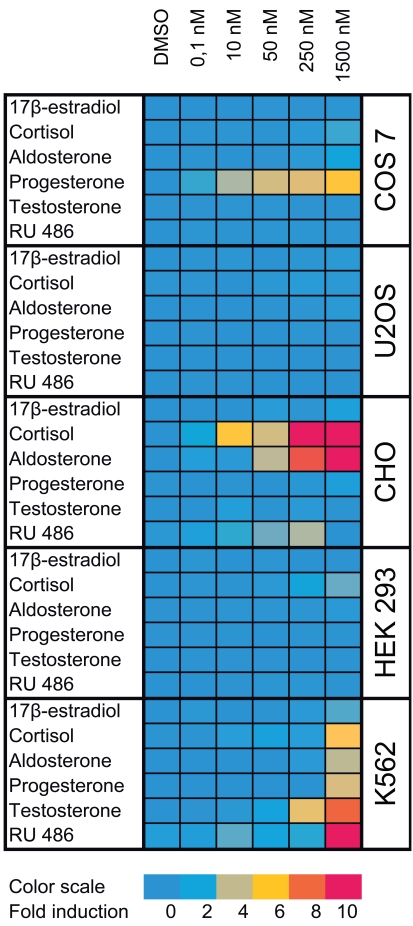
Activity of endogenous steroid receptors in commonly used cell lines. Selected cell lines (COS 7, U2OS, CHO, HEK 293 and K562) were transfected either with pGL4.26-3xERE [*luc2*/Hygro] or pGL4.26-3xGRE [*luc2*/Hygro] in the starvation medium and 24 hours after transfection cells were transferred to the white opaque 96-well plate at 2x10^4^ cells/well. Cells transfected with pGL4.26-3xERE [*luc2*/Hygro] were exposed to different concentrations of 17β-estradiol while cells transfected with pGL4.26-3xGRE [*luc2*/Hygro] were exposed to different concentrations of cortisol, aldosterone, progesterone, testosterone and mifepristone (RU 486) for another 24 hours. Control cells were exposed only to the diluent (DMSO). Luciferase activity was finally measured using ONE-Glo™ reagent (Promega) and fold induction was calculated as a ratio of the luciferase activity of cells treated with ligands and those treated with DMSO only. For clarity fold inductions are represented in the color scale.

Cross-talk with different signaling pathways is not the only technical problem that needs to be carefully considered during data analysis. Cross-talk with endogenously expressed steroid receptors can be even more important issue because ERα and ERβ bind to the same response elements, and GR, MR, AR and PR activate more or less efficiently promoters in both reporter vectors. When creating selective reporter cell lines, it is essential to express exogenous steroid receptors in cell lines with low background activity of other members of this receptor class. Unfortunately many of the cell lines traditionally used in the individual steroid receptor research do not fulfill this condition, and they could be only used in the study of small subset of receptors in the same time due to the endogenous expression of other steroid receptors. In order to create robust and selective reporter assays that would ideally be based on single parental cell line, we screened several commonly used cell lines for endogenous steroid receptor activity (Fig. **1**). Not surprisingly it was difficult to find a cell line with null background activity for all steroid receptors. For instance, COS7 cells derived from kidney of African green monkey and CHO cells from Chinese hamster ovary are often used in the study of ERs, which is in agreement with our observation that they have no ER activity. On the other hand COS7 cells are responsive to progesterone and CHO cells to cortisol and aldosterone when transfected with GRE containing reporter vector suggesting that they express nonnegligible levels of PR and GR/MR, respectively. In HEK 293 cells we found GR activity. No detectable activity of any steroid receptor was found in U2OS cells that was in agreement with previous observations [14]. In addition, these cells are easy to maintain and expand in cell culture and therefore are excellent for building up the panel of selective reporter cell lines.

As a starting point we have created four master cell lines stably transfected by following reporter vectors: pGL4.26-3xERE [*luc2*/Hygro], pGL4.26-3xGRE [*luc2*/Hygro], pGL4.36 [*luc2P*/MMTV/Hygro] and pGL4.35 [*luc2P*/9XGA L4UAS/Hygro]. These master cell lines were later transfected with corresponding expression vectors and the resulting reporter cell lines established after selection of resistant clones with antibiotics are listed in the Table **1**(supplementary material). To make sure that all cell lines are stable and do not change properties such as dynamic range or potency when exposed to ligands, they were cultured in the antibiotics containing medium for at least two months before freezing them and using them for compound profiling.

### Compound Profiling

**Fig. (2):**
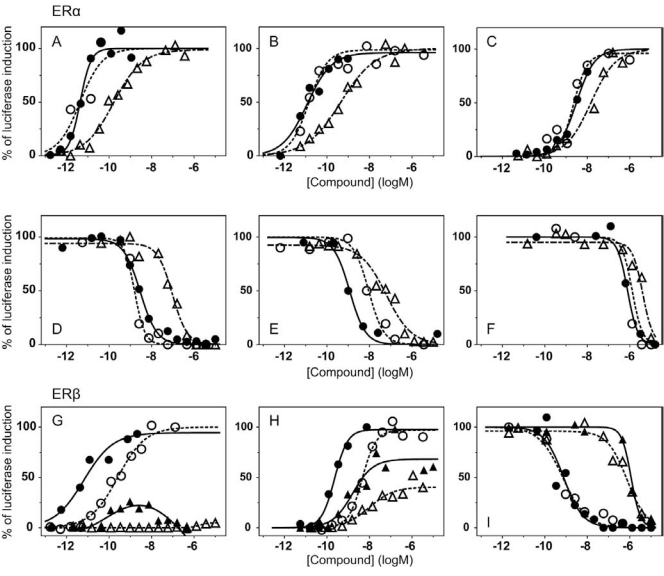
Transcriptional response of ER luciferase reporter cell lines to selected compounds. Different responses of ER luciferase reporter cell lines to selected compounds were obtained by the incubation of the cells with increasing concentrations of tested compounds for 20 hours. At the end of this time luciferase activity was measured. Values are expressed as a percentage of the maximal activation of the reporter system by E2. U2OS-ERα/3xERE (●), U2OS-ERα^wt^-LBD/9xGal4UAS (○) and U2OS-ERα^G420C^-LBD/9xGal4UAS cells (∆) were tested with E2 (**A**), DES (**B**) and PPT (**C**) in the agonist mode and with ICI 182.780 (**D**), raloxifene hydrochloride (**E**) and tamoxifen citrate (**F**) in the presence of 1 nM E2 for U2OS-ERα/3xERE, 1 nM E2 for U2OS-ERα^wt^-LBD/9xGal4UAS and 20 nM E2 for U2OS-ERα^G420^C- LBD/9xGal4UAS in the antagonist mode. (**G**) U2OS-ERβ/3xERE reporter cell line was tested with E2 (●) and PPT (▲); U2OS-ERβ- LBD/9xGal4UAS with E2 (○) and PPT (∆). (**H**) U2OS-ERβ/3xERE was tested with DPN (●) and genistein (▲); U2OS-ERβ- LBD/9xGal4UAS with DPN (○) and genistein (∆). (**I**) Antagonistic response of U2OS-ERβ/3xERE reporter cell line to raloxifene hydrochloride (●) and to tamoxifen citrate (▲)in the presence of 1 nM E2 and the response of U2OS-ERβ-LBD/9xGal4UAS reporter cell line to raloxifene hydrochloride (○) and to tamoxifen citrate (∆) in the presence of 5 nM E2.

**Fig. (3):**
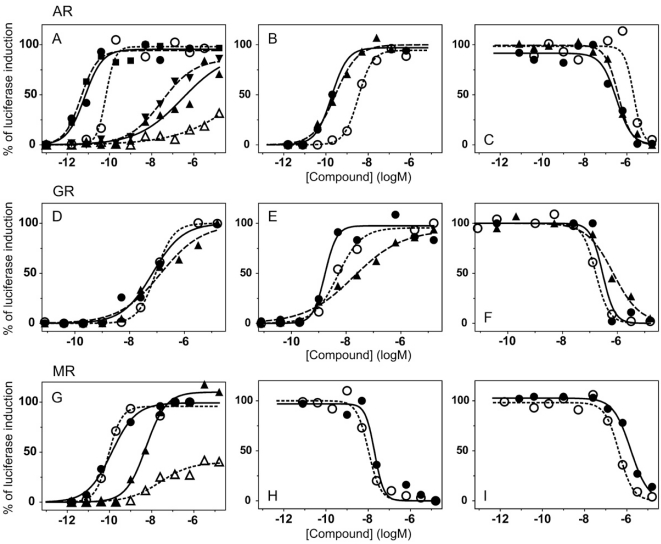
Transcriptional response of AR, GR and MR luciferase reporter cell lines to selected compounds. Different responses of AR, GR and MR luciferase reporter cell lines to selected compounds were obtained by the incubation of the cells with increasing concentrations of tested compounds for 20 hours. At the end of this time luciferase activity was measured. (**A**) U2OS-AR/3xGRE cells were tested with dihydrotestosterone (●) and E2 (▲); U2OS-AR/MMTV with dihydrotestosterone (■) and E2 (▼); U2OS-AR-LBD/9xGal4UAS with dihydrotestosterone (○) and E2 (∆). (**B**) Agonistic response of U2OS-AR/3xGRE (●), U2OS-AR/MMTV (▲) and U2OS-ARLBD/ 9xGal4UAS (○) reporter cell lines to testosterone. (**C**) Antagonistic response of U2OS-AR/3xGRE (●), U2OS-AR/MMTV (▲) and U2OS-AR-LBD/9xGal4UAS (○) reporter cell lines to nilutamide in the presence of 0.05 nM, 0.05 nM and 1 nM dihydrotestosterone respectively. U2OS-GR/3xGRE (●), U2OS-GR/MMTV (▲) and U2OS-GR-LBD/9xGal4UAS (○) reporter cell lines were tested with cortisol (**D**) and betamethasone (**E**) in the agonist mode and with cyproterone acetate in the presence of 5 nM dexamethasone for all of the reporter cell lines (**F**). (**G**) U2OS-MR/MMTV reporter cell line was tested with aldosterone (●) and dexamethasone (▲); U2OS-MRLBD/ 9xGal4UAS with aldosterone (○) and dexamethasone (Δ). Antagonistic response of U2OS-MR/MMTV (●) and U2OS-MRLBD/ 9xGal4UAS (○) reporter cell lines to spironolactone (**H**) and to dihydrotestosterone in the presence of 1 nM aldosterone for all of the reporter cell lines (**I**).

To validate these cell lines with respect to their utility to detect agonistic and antagonistic properties of compounds, we used them for profiling a set of 28 well-established ligands, comprising both natural ligands and synthetic compounds, commonly used in clinics or as potent tools in the study of steroid receptors. The profiling was performed in the agonist and antagonist mode, and LogEC_50_/LogIC_50_ values calculated from dose response curves (Figs. **2**, **3** and data not shown) are summarized in the Table **1**.

### Characterization of Full-Length Steroid Receptor Reporter Cell Lines

Generally all U2OS reporter cell lines expressing full-length steroid receptors were sensitive to their commonly used agonists and antagonists. EC_50_ values varied between 1 pM and 100 pM for the following combinations ERα/E2, ERβ/E2, AR/DHT and MR/aldosterone and the potency of GR-expressing reporter cell lines was close to 1 nM for dexamethasone: EC_50_ (U2OS-GR/3xGRE) = 1.66 nM and EC50 (U2OS-GR/MMTV) = 1.03 nM (Table **1e**). The lower potency of dexamethasone and glucocorticoids more generally on GR (Fig. **3d**, Table **1e**) in comparison to potencies observed in the 17β-estradiol induced transactivation by ERα and ERβ (Fig. **2a**, **g** and Table **1a**) or dihydrotestosterone induced transactivation by AR (Fig. **3a** and Table **1c**) was expected and is consistent with data in the literature [19]. GR generally shows slightly lower affinity to its ligands compared to other steroid receptors. ERα and ERβ reporter cell lines showed high sensitivity to estrogenic compounds as illustrated by 17β-estradiol with potency attacking picomolar range: EC_50_(U2OS-ERα/3xERE) = 4 pM and EC_50_(U2OS-ERβ/3xERE) = 5 pM (Table **1a**). Similarly both AR reporter cell lines respond to dihydrotestosterone in the picomolar range: EC_50_(U2OS-AR/3xGRE) = 4 pM and EC_50_(U2OS-AR/MMTV) = 3 pM (Table **1c**) and MR to aldosterone in the subnanomolar range: EC50(U2OS-MR/MMTV) = 88 pM (Table **1g**). We also compared data generated with our cell lines to values in the literature and found our data comparable to literature reports for all receptor/promoter combinations [11, 12, 14, 19-21].

### Compound Selectivity

The same cellular background represented by U2OS cell type devoid of any endogenous steroid receptor activity enabled the construction of a consistent reporter system allowing monitoring activity of unique receptors with exceptional selectivity. Our data confirm this premise and the selectivity is illustrated by the fact that we did not find any sign of luciferase activity when treating ERα and ERβ reporter cell lines with glucocorticoid activity-exhibiting compounds (dexamethasone, beclomethasone, betamethasone, triamcinolone, cyproterone acetate etc.) even at 10 μM concentration.

Similarly no agonistic activity was observed with GR and MR reporter cell lines treated with estrogens or androgens (Table **1e**, **g**). Since these receptors are generally inhibited by estrogens, androgens and progestagens at higher concentrations, we detected their antagonistic activity especially in MR reporter cell lines. In the tested concentration range they can be ranked according their decreasing antagonistic potency to MR: progesterone > testosterone > dihydrotestosterone > 17β-estradiol > danazol, PPT.

On the other hand we measured a weak partial agonism of glucocorticoids on both full-length AR reporter cell lines with MMTV and GRE containing promoter. The activation efficacy was lower than 10% of the full activation of the receptor by dihydrotestosterone in most cases and never exceeded 20% (data not shown). In addition it was stronger than in the master reporter cell lines U2OS-/3xGRE and U2OS-/MMTV, and we did not detect the same activity with U2OS-AR-LBD / 9xGal4UAS cell line, which is however less sensitive as discussed below. Therefore we hypothesize that the weak androgenic activation by glucocorticoids results from mixed activity of very low level of endogenously expressed GR and cross reactivity of glucocorticoids with AR.

### Comparison of 3xGRE Reporters Versus MMTV

**Fig. (4):**
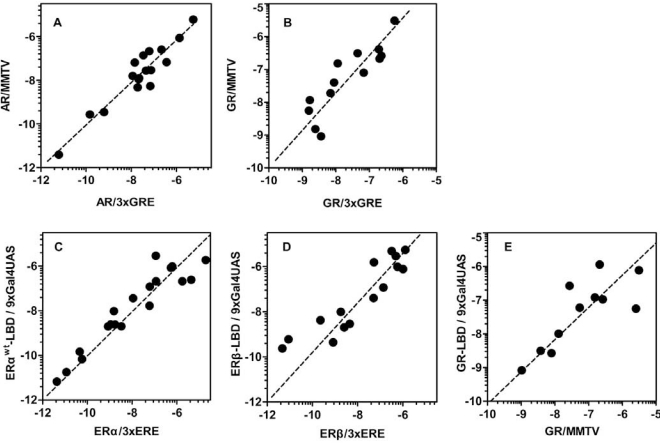
Comparison of different reporter formats and promoters. The effect of the different promoter use in the full-length AR (**A**) and GR (**B**) reporter assays was analyzed by correlating LogEC_50_ and LogIC_50_ values of compounds active in the reporter assays containing GRE in the reporter promoter and the same values obtained from reporters with MMTV promoter. Pearson correlation coefficient was calculated: AR (Pearson r coefficient = 0.95, r^2^ = 0.89, P < 0.0001), GR (Pearson r coefficient = 0.88, r^2^ = 0.77, P < 0.0001). Correlation of compound potencies obtained from different reporter formats. LogEC_50_ together with LogIC50 values of compounds active in the ERα (**C**), ERβ (**D**) and GR (**E**) full-length reporter assays were plotted against the same values obtained from Gal4/LBD reporter assays. Pearson correlation coefficient was calculated: ERα (Pearson r coefficient = 0.94, r^2^ = 0.89, P < 0.0001), ERβ (Pearson r coefficient = 0.91, r^2^ = 0.82, P < 0.0001), GR (Pearson r coefficient = 0.80, r^2^ = 0.63, P =0.0034).

To estimate to what extent promoter context contributes to different responses provoked by the same ligand and receptor we compared compound potencies from reporter cell lines which contain either synthetic promoter containing 3xGRE or more complex viral promoter from MMTV. Two AR reporter cell lines show almost identical response to tested compounds considering the potency and efficacy of the dose response (r^2^=0.89, Fig. **4a**). Generally it can be noticed that U2OS-AR/MMTV is slightly more sensitive than U2OS-AR/3xGRE with some dose response curves shifted slightly to lower concentrations. Both cell lines show the same mixed agonistic/antagonistic profiles for cyproterone acetate, progesterone and mifepristone with comparable partial activities (Table **1c**, **1d**).

Very similar profiles were also obtained with GR reporter cell lines (r^2^=0.77, Fig. **4b**). We could identify few different responses in the antagonist mode where U2OS-GR/MMTV was less sensitive to detect the antagonistic properties of some compounds like spironolactone or mifepristone. However, U2OS-GR/MMTV cell line provides about three times higher dynamic range and better data reproducibility (data not shown).

### Comparison of Full-Length Versus Gal4/LBD Reporter Formats

Since steroid receptors are complex molecules with complicated organization of the molecule one of the most important concerns is that removal of the N-terminal part of the steroid receptor molecule changes considerably the response to compounds. In order to see whether the Gal4/LBD reporter cell lines respond in the same way or differently to tested compounds we carefully analyzed potencies and partial/full activities obtained from both of these formats.

### ERα

For ERα we have prepared two Gal4/LBD reporter cell lines: U2OS-ERα^wt^-LBD /9xGal4UAS and U2OS-ERα^G420C^-LBD /9xGal4UAS. The second reporter cell line was described recently [13] and carries one amino acid substitution causing higher fold induction of the luciferase activity compared to wild type ERα-LBD (data not shown) but also decreases the affinity of ligands to receptors by 0.5 – 1.5 logs depending on the particular ligand (Table **1a** and **1b**). There is very little difference between the response from U2OS-ERα/3xERE and U2OS-ERα^wt^-LBD in both agonist and antagonist mode as illustrated by few examples in the Fig. (**2a-f**) and high correlation of LogEC50, LogIC50 values obtained with these two different reporter formats (r^2^=0.89, Fig. **4c**). An exception is probably somewhat consistently higher potency of androgenic compounds in ERα-LBD reporters about 1 log but these compounds activate ERα in the micromolar and higher concentration range.

### ERβ

The response to compounds mediated by ERβ-LBD is shifted of about 1.5 logs to higher concentrations compared to full-length ERβ reporter (Fig. **4d**, Table **1a**). This shift is noticeable mainly in the agonist mode and is consistent in the whole compound collection; hence the rank of potencies is conserved between LBD and full-length ERβ reporters. It is worth noting that ERβ is considerably weaker transactivator than ERα due to lower affinity for ERE half-sites [22-24]. In agreement with this observation, we obtained only moderate fold induction of luciferase activity using U2OS-ERβ/3xERE cell line (3 to 4-fold) in contrast to wider dynamic range offered by U2OS-ERβ-LBD/9xGal4UAS cell line (25-fold).

### GR and MR

With GR and MR both formats gave similar profiles with tested compounds in the agonist mode as shown in Table **1e**, **1g** and Fig. (**3d-i**). When looking closely at data from both formats of GR reporters, we concluded that U2OS-GR-LBD/9xGal4UAS cell line generally tends to be more sensitive to antagonist effect of compounds. For example mifepristone is partial agonist/full antagonist on both full-length GR reporter cell lines but very potent pure full antagonist on LBD format. Also we didn´t detect very weak partial agonistic activity of progesterone using U2OS-GR-LBD/9xGal4UAS. Similarly, MR-LBD expressing reporter cell line is in contrast to its full-length MR counterpart only weakly and partially activated by glucocorticoids such as dexamethasone, betamethasone and beclomethasone, and interestingly these compounds have pronounced full antagonist effect in the LBD format, while there is no sign of antagonistic activity in the full-length receptor format. The correlation between (LogEC50, Log IC50) obtained from different formats of GR reporters is lower than in case of ERα or ERβ mainly due to the artifacts in the antagonist mode (r^2^=0.63, Fig. **4e**). These data suggest that MR-LBD and GR-LBD based reporter cell lines generally reproduce well the activity profiles of tested compounds as obtained using reporter cell lines expressing full-length receptors but in addition can produce false positives when assayed in the antagonist mode.

### AR

It was previously shown that the main transactivation activity inducible by ligand resides in AF-1 of the N-terminus of the AR molecule instead of AF-2 [25-27] and this property makes AR unique in the steroid receptor family. Unfortunately this property of AR makes it also difficult to prepare an assay where only C-terminal part of the molecule containing LBD with AF-2 is used. When expressing the chimeric AR-LBD fused to Gal4-DBD in U2OS cells we were almost not able to detect any androgen-induced luciferase activity from the reporter vector. Since the ligand-induced activation of AR requires the interaction between AF-1 and AF-2 [28, 29], we speculated that the co-expression of the full-length AR would be sufficient for creating a functional AR-LBD containing transactivation complex able to induce transcription from the reporter vector containing Gal4 UAS sites. This hypothesis was confirmed in transient transfections and later by preparation of the stable reporter cell line U2OS-AR-LBD/9xGal4UAS by cotransfecting pBIND-AR construct together with pcDNA3-hAR vector. This cell line however showed decreased sensitivity to tested compounds as illustrated by Fig. (**3a**, **b**, **c**) and Table **1c**. The dose response curve is shifted in average about 1 log to higher concentrations for high affinity ligands. We also noticed that the dose response profile of low affinity ligands diverged more substantially from profiles obtained by full-length AR reporter cell lines as illustrated by partial activation of AR-LBD by E2 (Fig. **3a**) and other compounds like spironolactone, cyproterone acetate and danazol.

## DISCUSSION AND CONCLUSION

We have created and subsequently characterized two panels of U2OS-based luciferase steroid receptor reporter cell lines using two different reporter formats: Gal4/LBD reporter cell lines and full-length steroid receptor reporter cell lines. We have characterized these cell lines by profiling them using a set of 28 well established ligands and analyzed their properties in terms of compound potency, efficacy, selectivity and reproducibility of results among different formats.

The main advantage of the whole system is that it is based on single cell type: osteosarcoma U2OS cell line, and therefore it has unified and constant cellular background that is defined by the expression of the same type and amounts of enzymes involved in the metabolism of compounds and the same coactivators and corepressors regulating the steroid receptor transcription response. Thus results obtained from different reporter cell lines can be directly compared. This is also closely related to the selectivity of cell lines and to the question to what extent is the compound activity mediated by one given receptor. The use of uniform cellular background makes the estimation of contribution of different receptors to the measured compound activity easier since it is possible to test the compound on all receptors and compare with values from master reporter cell lines carrying the reporter vector alone. In addition we have shown that U2OS cells have no detectable activity of any of the steroid receptors and therefore there is no cross-talk with endogenously expressed receptors. This was demonstrated by showing selective activation of specific receptors when exposed to selective ligands. Taken together and considering the fact that U2OS cells are robust and easy to maintain in culture, these properties makes the whole reporter system highly consistent, selective and robust.

As a next step we have carefully compared and analyzed data obtained using both assay formats. The advantage of full-length steroid receptor reporter system is that it is based on the native fully functional receptors that can bind to high affinity response elements in the promoter of the reporter vectors. Because some receptors interact with the DNA and change their conformation according to the response element, we have also prepared reporter cell lines for AR, GR and MR where the defined response element (3xGRE) was replaced by the complex viral promoter derived from the MMTV LTR. Although we have noticed subtle differences between the cell lines using MMTV instead of 3xGRE, generally the use of MMTV provided the same information about the compound activity, improved dynamic range of the assay and reproducibility of data but also increased the risk of integrating signals from other signaling pathways *via *binding sites for different cellular transcription factors.

The problem of using overexpression of the fully biologically active full-length steroid receptors is potential lower tolerance of higher expression levels by the cells. As a result cells expressing higher levels of the specific receptor are usually unstable in the culture and the dynamic range of the assay might be limited from this reason.

Another problem with full-length receptors is that they respond to multiple stimuli from within the cell, and their activity can be modulated indirectly by posttranslational modifications mainly on the N-terminal part of the molecule as a result of activation of the other signaling pathways such as MAPK and PKA, for example.

These problems are not associated with the Gal4/LBD reporter format since the activity is mediated through Gal4/UAS response elements foreign to most of the mammalian transcription factors, and consequently this format has generally low background activity. Moreover the N-terminal AF-1 with regulatory sequences was removed from the chimeric receptors, and therefore the connection between the biological activity of the compound and the measured luciferase activity is more tightly associated with the specific activity of the steroid receptor as a result of direct compound binding.

The comparison of compound profiles from Gal4/LBD reporter assays with those from full-length receptor reporter assays showed that there is a high consensus between these two formats especially for ERα, ERβ and GR. Although some Gal4/LBD reporter cell lines suffer from slightly decreased potency (0.5-1.5 logs) like U2OS-ERα^G420C^-LBD/9xGal4UAS, U2OS-ERβ-LBD/9xGal4UAS and U2OS-AR-LBD/9xGal4UAS the rank of potencies of tested compounds is surprisingly well conserved. We have however identified few weak points of this format in U2OS-GR-LBD/9xGal4UAS and U2OS-MR-LBD/9xGal4UAS. Inactive or purely agonistic compounds in the full-length format tended to show antagonistic properties when assayed with U2OS-GR-LBD/9xGal4UAS. The effect was even more pronounced with U2OS-MR-LBD/9xGal4UAS where some compounds exhibited both decreased potency and efficacy in the agonist mode and became full antagonists in the opposite mode. In other words MR-LBD and GR-LBD-based reporters generally reproduce the activity profiles of some compounds compared to full-length receptor reporters, but can produce false positives when tested in the antagonist mode.

On the other hand Gal4/LBD reporters reflected surprisingly reliably some subtle activity characteristics of compounds such as partial agonism. For example genistein, ERβ selective isoflavone was almost full agonist in U2OS-ERα/3xERE assay, partial agonist in U2OS-ERβ/3xERE assay, full agonist in both U2OS-ERα^G420C^-LBD/9xGal4UAS and U2OS-ERβ-LBD/9xGal4UAS assay and partial agonist in U2OS-ERβ-LBD/9xGal4UAS assay reflecting clearly and perfectly the response from full-length receptors (data not shown). Similarly weak partial agonism of selective estrogen receptor modulators (SERMs) such as 4-hydroxy-tamoxifen, tamoxifen citrate or raloxifene hydrochloride was also detected using Gal4/LBD assays although it was less pronounced than in full-length receptor reporter format (Table **1a**).

The expression of chimeric receptors is generally well tolerated by the host cell and considerably improves the dynamic range of the reporter assays compared to its full-length receptor counterpart with one exception being AR, where the removal of N-terminus causes almost complete silencing of the ligand induced transactivation as discussed above.

From these reasons Gal4/LBD reporter format is especially well suited for HTS campaigns. We have successfully carried out the compound profiling using all reporter cell lines in the 384-well format using instruments for HTS (Pin tool robotic compound transfer). In order to assess the reproducibility of the recorded data we have calculated Z´-factor for all the reporter cell lines in the Gal4/LBD reporter format (Table , **1b**, supplementary material). Z´-factor exceeding 0.5 is commonly accepted criterion for the satisfactory assay quality for the HTS [30]. Four out of eight Gal4/LBD reporter assays exhibit Z´-factor in the range of 0.76 and 0.80 which is a sign of an excellent cell-based assay very well adapted to HTS. Only two reporter assays showed Z´-factor lower than 0.7: U2OS-ERα^wt^-LBD/9xGal4UAS and U2OS-AR-LBD/9xGal4UAS. The relatively low fold induction of the ERα^wt^-LBD reporter assay causing lower Z´-factor (0.65) was mentioned above and can be overcome by the introduction of the G420C mutation to the ERα molecule (full-length receptor amino acid numbering). The resulting reporter assay (U2OS-ERα^G420C^-LBD/9xGal4UAS) has 10 times higher fold induction (data not shown) and higher Z´-factor (0.76). In addition the Gal4/LBD reporter assays can benefit from the constitutive expression of a *Renilla *luciferase-neo^R^ fusion gene which can be used as an internal control in the HTS and can be helpful in compensating for variability between samples caused by difference in cell number for example by random pipetting errors or by nonspecific interference with the luciferase reporter readout and cytotoxic effects of compounds [13].

Because each of these formats represents specific set of advantages and drawbacks, altogether they embody a powerful and complete tool for profiling of large compound libraries and identifying modulators showing interesting and useful activity profiles over the whole family of steroid hormone receptors. They are also sophisticated tools for the detailed study of mechanisms by which these compounds modulate the activity of the specific steroid receptors.

## SUPPLEMENTARY MATERIAL

Supplementary material is available on the publishers Web site along with the published article.

## Figures and Tables

**Table 1a:** 

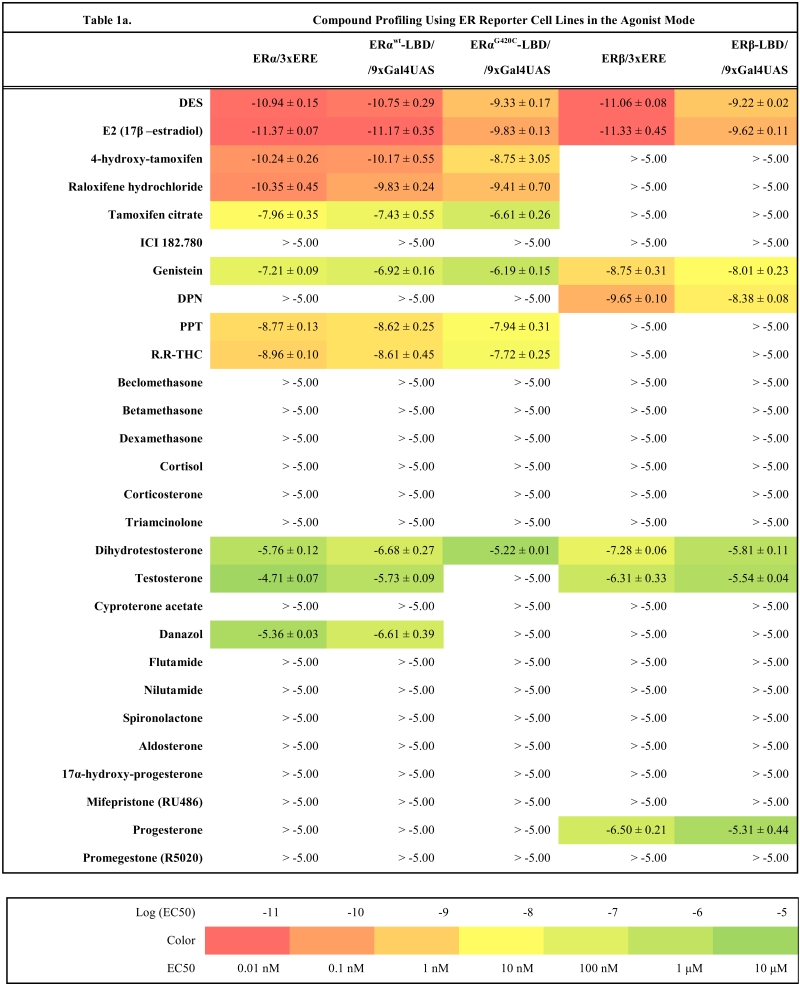

**Table 1b:** 

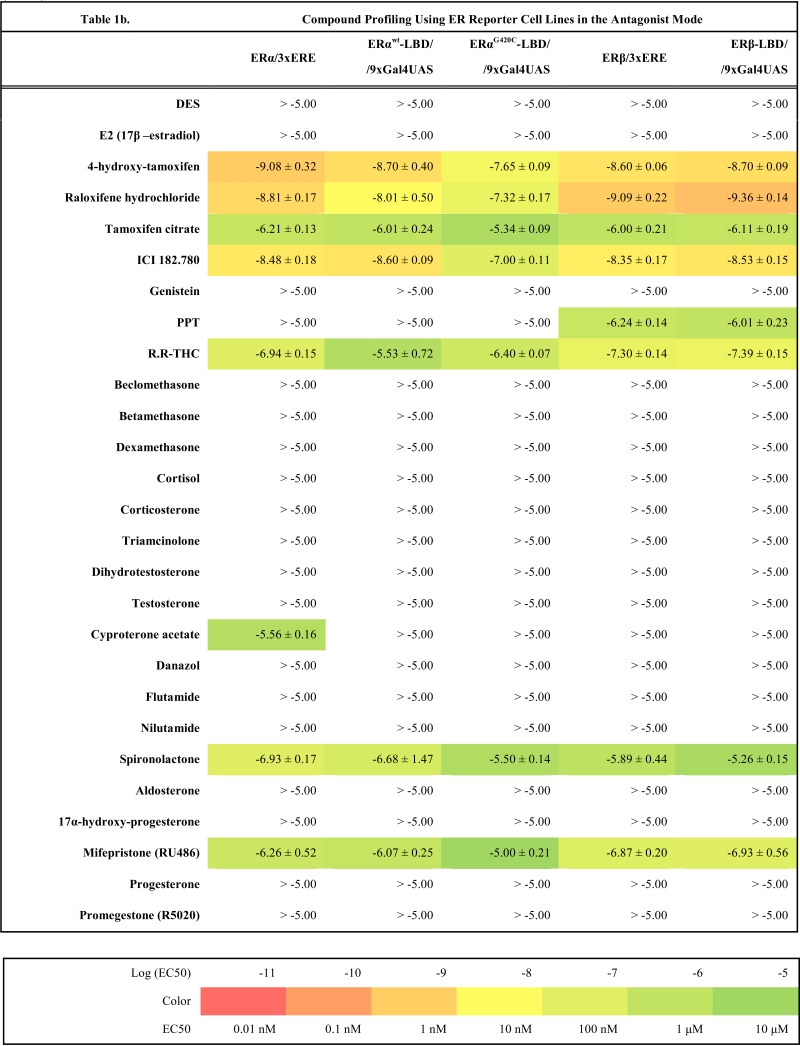

**Table 1c:** 

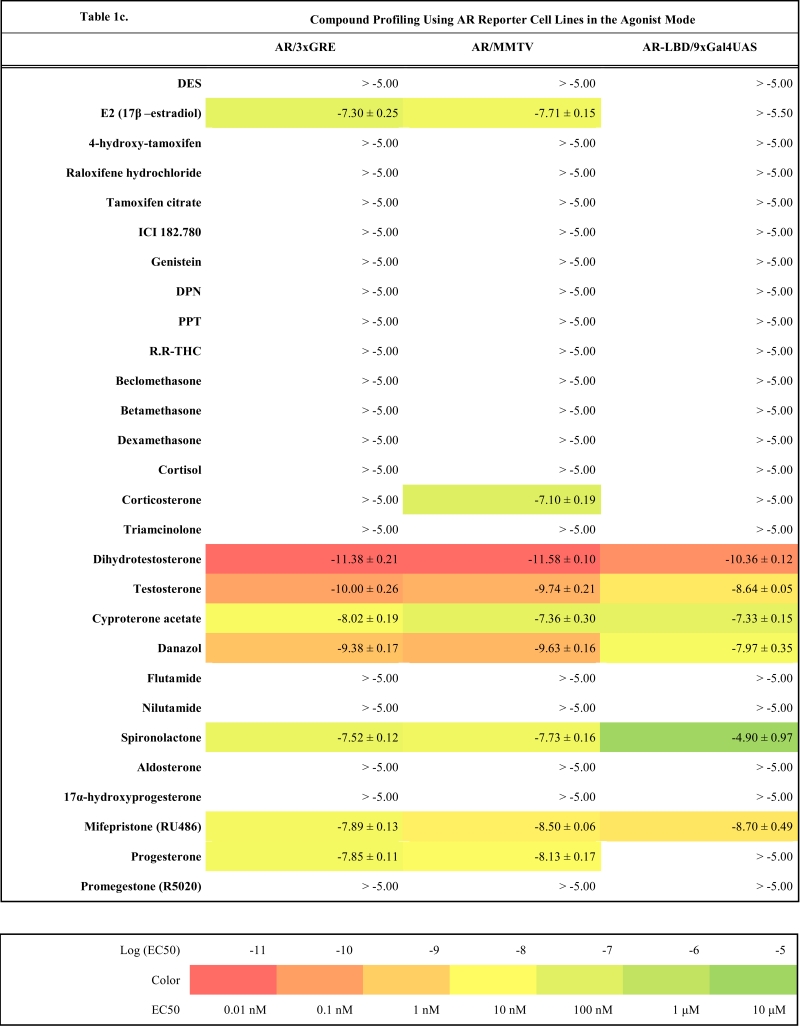

**Table 1d:** 

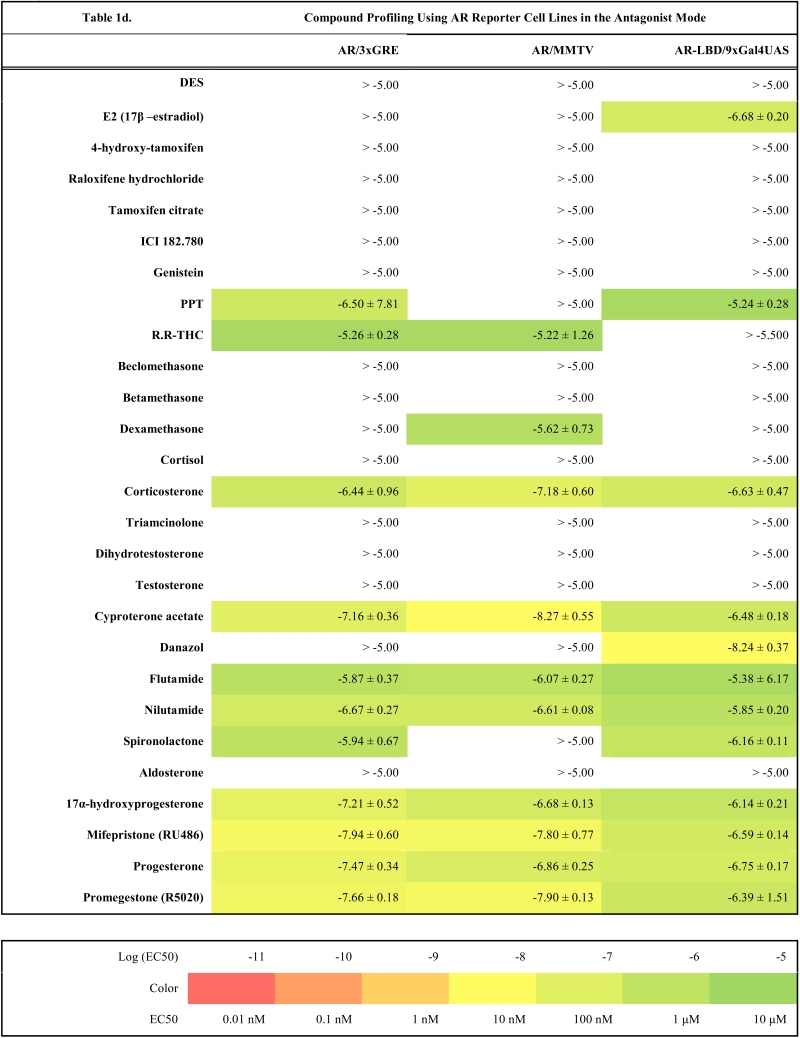

**Table 1e:** 

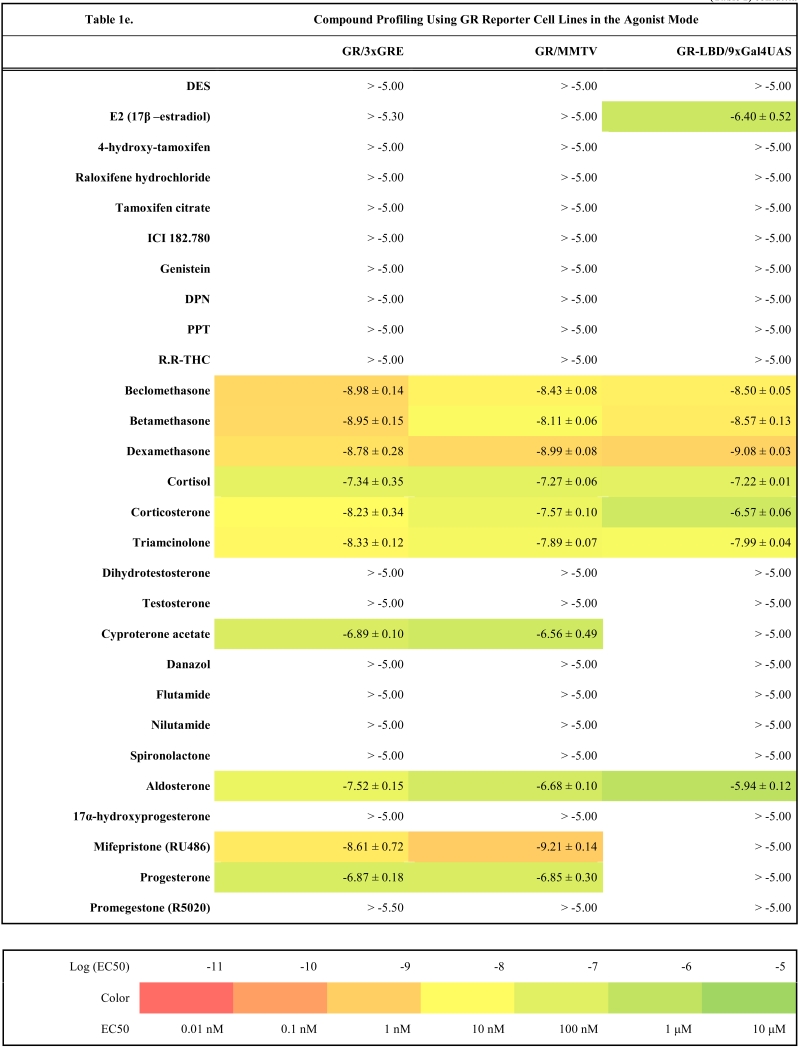

**Table 1f:** 

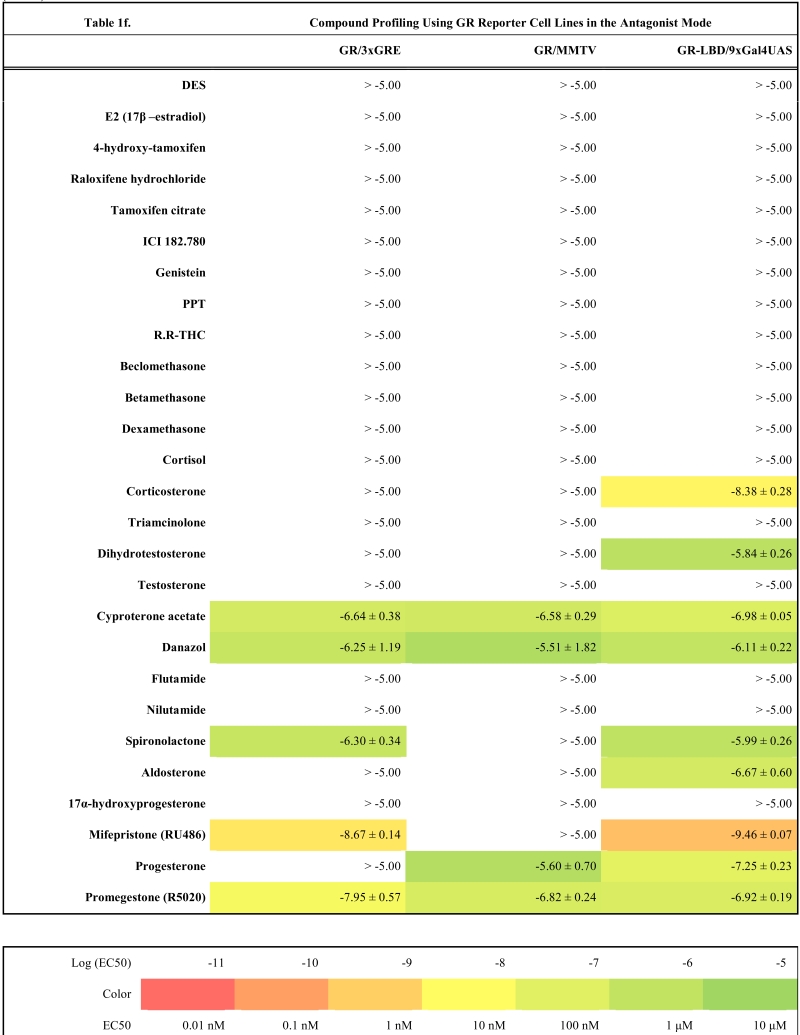

**Table 1g:** 

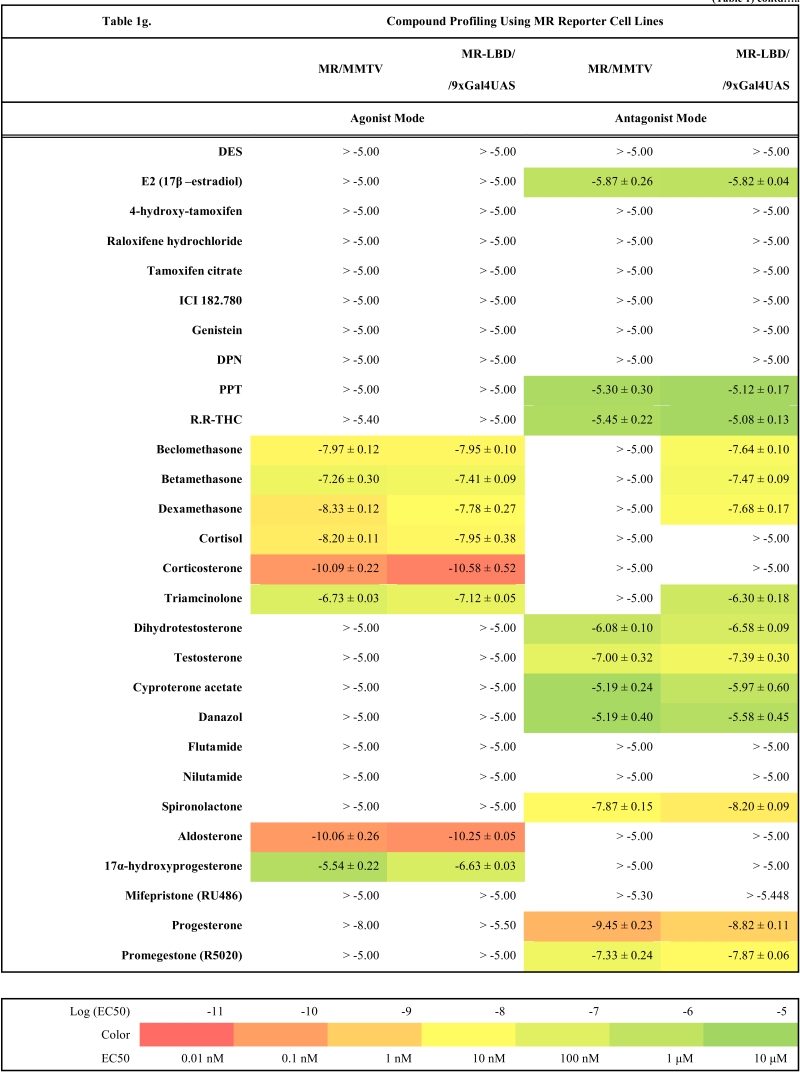

**Table 1h:**