# Supplementary material for: Two Panels of Steroid Receptor Luciferase Reporter Cell Lines for Compound Profiling
Source: Comb Chem High Throughput Screen. 2011 May;14(4):248–66. doi: 10.2174/138620711795222446 (PMC3234597; doi:10.2174/138620711795222446)
Supplement: Supplementary file 1 [file CCHTS-14-248_SD1.pdf]

SUPPLEMENTARY MATERIAL

Two Panels of Steroid Receptor Luciferase Reporter Cell Lines for Compound Profiling

David Sedlák[1], Aileen Paguio[2] and Petr Bartůněk[*,1]

[1]*Center for Chemical Genetics, Institute of Molecular Genetics, v.v.i., Academy of Sciences of the Czech Republic, Vídeňská 1083, 142 20 Prague, Czech Republic*

[2]*Promega Corporation, 2800 Woods Hollow Road Madison, WI 53711 USA*

Table 1. List of Full-Length Steroid Receptor (a) and LBD/Gal4UAS (b) Reporter Cell Lines Established in U2OS Cells by Transfection of the Listed Plasmid Combinations and by Subsequent Selection of Stable Transfectants. Z´-Factor was Determined Using the Following Formula $Z' = 1-[(3*SD_{treated} + 3*SD_{untreated})/ (Average_{treated} - average_{untreated})]$ in the Agonist Mode

Table 1a. Full-Length Steroid Receptor Reporter Cell Lines

Steroid Receptor	Expression Vector	Reporter Vector	Reporter Cell Line
-	-	pGL4.26-3xERE [*luc2*/Hygro]	**U2OS-/3xERE**
-	-	pGL4.26-3xGRE [*luc2*/Hygro]	**U2OS-/3xGRE**
-	-	pGL4.36 [*luc2P*/MMTV/Hygro]	**U2OS-/MMTV**
**ERα**	pcDNA3-hERα	pGL4.26-3xERE [*luc2*/Hygro]	**U2OS-ERα/3xERE**
**ERβ**	pcDNA3-hERβ	pGL4.26-3xERE [*luc2*/Hygro]	**U2OS-ERβ/3xERE**
**AR**	pcDNA3-hAR	pGL4.26-3xGRE [*luc2*/Hygro]	**U2OS-AR/3xGRE**
**AR**	pcDNA3-hAR	pGL4.36 [*luc2P*/MMTV/Hygro]	**U2OS-AR/MMTV**
**GR**	pcDNA3-hGR	pGL4.26-3xGRE [*luc2*/Hygro]	**U2OS-GR/3xGRE**
**GR**	pcDNA3-hGR	pGL4.36 [*luc2P*/MMTV/Hygro]	**U2OS-GR/MMTV**
**MR**	pcDNA3-hMR	pGL4.36 [*luc2P*/MMTV/Hygro]	**U2OS-MR/MMTV**

Table 1b. Steroid Receptor LBD Reporter Cell Lines

Steroid Receptor	Expression Vector	Reporter Vector	Reporter Cell Line	Z´-Factor Agonist Mode
-	-		**U2OS-/9xGal4UAS**	-
**ERα**	pBIND-ERα^{wt}		**U2OS-ERα^{wt}-LBD /9xGal4UAS**	0.65
**ERα**	pBIND-ERα^{G420C}		**U2OS-ERα^{G420C}-LBD /9xGal4UAS**	0.76
**ERβ**	pBIND-ERβ	pGL4.35 [luc2P/9XGAL4UAS /Hygro]	**U2OS-ERβ-LBD /9xGal4UAS**	0.72
**AR**	pBIND-AR		**U2OS-AR-LBD / 9xGal4UAS**	0.67
**GR**	pBIND-GR		**U2OS-GR-LBD / 9xGal4UAS**	0.80
**MR**	pBIND-MR		**U2OS-MR-LBD / 9xGal4UAS**	0.78
**PR**	pBIND-PR		**U2OS-PR-LBD / 9xGal4UAS**	0.78

Table 2. List of 28 Compounds Used in the Profiling of the Panel of Steroid Receptor Reporter Cell Lines, their Structure, Origin and Known Biological Activity.

Compound	Structure	Supplier	Known Biological Activity
**DES**	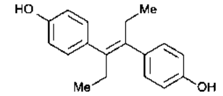	Sigma-Aldrich	ERα, ERβ agonist
**E2 (17β –estradiol)**	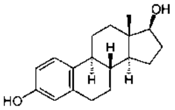	Sigma-Aldrich	ERα, ERβ agonist
**4-hydroxy-tamoxifen**	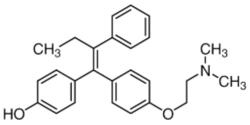	Sigma-Aldrich	SERM
**Raloxifene hydrochloride**	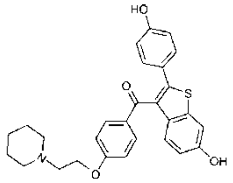	Sigma-Aldrich	SERM
**Tamoxifen citrate**	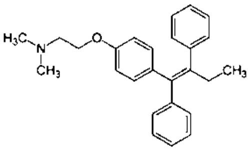	Sigma-Aldrich	SERM
**ICI 182.780**	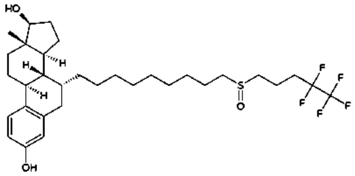	Sigma-Aldrich	ERα, ERβ full antagonist
**Genistein**	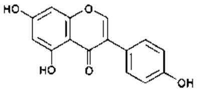	Sigma-Aldrich	ERβ selective agonist
**DPN**	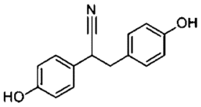	Tocris Bioscience	ERβ selective agonist
**PPT**	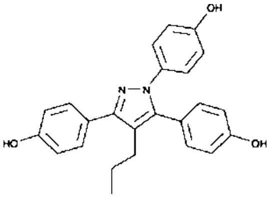	Tocris Bioscience	ERα selective agonist
**R.R-THC**	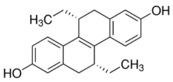	Sigma-Aldrich	ERα agonist, ERβ antagonist
**Beclomethasone**	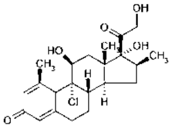	Sigma-Aldrich	GR agonist

(Table 2) contd…..

Compound	Structure	Supplier	Known Biological Activity
**Betamethasone**	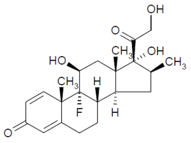	Sigma-Aldrich	GR agonist
**Dexamethasone**	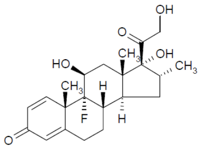	Sigma-Aldrich	GR agonist
**Cortisol**	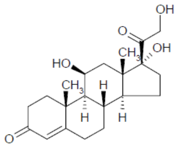	Sigma-Aldrich	GR agonist
**Corticosterone**	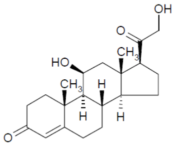	Sigma-Aldrich	GR agonist
**Triamcinolone**	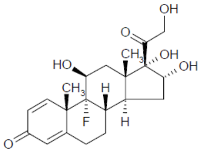	Sigma-Aldrich	GR agonist
**DHT (dihydrotestosterone)**	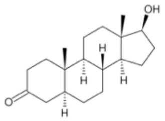	Sigma-Aldrich	AR agonist
**Testosterone**	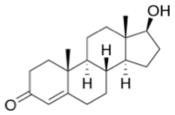	Sigma-Aldrich	AR agonist
**Cyproterone acetate**	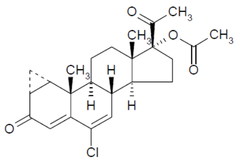	Sigma-Aldrich	AR antagonist
**Danazol**	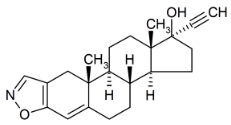	Sigma-Aldrich	AR agonist
**Flutamide**	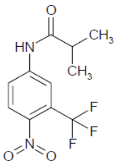	Sigma-Aldrich	AR antagonist
**Nilutamide**	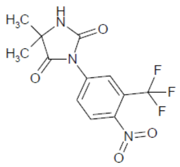	Sigma-Aldrich	AR antagonist

(Table 2) contd…..

Compound	Structure	Supplier	Known Biological Activity
**Spironolactone**	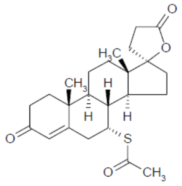	Sigma-Aldrich	MR antagonist
**Aldosterone**	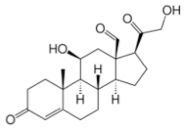	Sigma-Aldrich	MR agonist
**17α-hydroxy-progesterone**	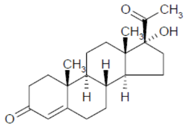	Sigma-Aldrich	low activity on steroid receptors
**Mifepristone (RU486)**	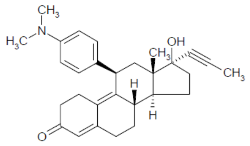	Sigma-Aldrich	PR antagonist
**Progesterone**	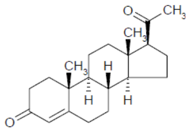	Sigma-Aldrich	PR agonist
**Promegestone (R5020)**	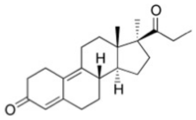	PerkinElmer	PR agonist
